# Cardiac safety of afatinib: a review of data from clinical trials

**DOI:** 10.1186/s40959-015-0006-7

**Published:** 2015-11-26

**Authors:** Michael S. Ewer, Kalpesh Patel, Dennis O’Brien, Robert M. Lorence

**Affiliations:** 1 Department of Cardiology, Division of Medical Specialties, The University of Texas MD Anderson Cancer Center, 1515 Holcombe Blvd., Houston, TX 77030 USA; 2 Boehringer Ingelheim Pharmaceuticals, Inc., Ridgefield, CT USA

**Keywords:** Afatinib, Cardiac safety, Non–small cell lung cancer, Tyrosine kinase inhibitor

## Abstract

**Background:**

Afatinib is an oral irreversible ErbB family blocker that targets epidermal growth factor receptor (EGFR/ErbB1), human epidermal growth factor receptor 2 (HER2/ErbB2), and HER4 (ErbB4) and is approved for the first-line treatment of advanced non–small cell lung cancer (NSCLC) with certain sensitizing *EGFR* mutations. As anti-HER2 therapies have been associated with cardiac dysfunction, we report cardiac safety data for afatinib.

**Methods:**

Cardiac data were analyzed from phase III trials of afatinib 40 mg in treatment-naive patients with *EGFR* mutation–positive NSCLC (LUX-Lung 3 [LL3]; *n* = 229 afatinib, *n* = 111 chemotherapy) and afatinib 50 mg in EGFR tyrosine kinase inhibitor–pretreated NSCLC patients (LUX-Lung 1 [LL1]; *n* = 390 afatinib, *n* = 195 placebo). Additional pooled data from 49 trials (*n* = 3865 afatinib-treated patients) is reported. Cardiac failure adverse events (CF-AEs), including symptomatic cardiac failure and depressed left ventricular ejection fraction (LVEF), were analyzed.

**Results:**

Time at risk–adjusted CF-AE rates (events/100 patient-years) were similar for afatinib versus placebo in LL1 (2.40 vs 2.23) and versus chemotherapy in LL3 (2.28 vs 2.92); the pooled afatinib CF-AE rate (2.88) was consistent with that for both trials. The frequency of clinically significant LVEF reductions was higher for chemotherapy in LL3 (2/15 [13.3 %], afatinib 13/208 [6.3 %]; *p* = 0.267) and similar to placebo in LL1 (5/122 [4.1 %], afatinib 14/304 [4.6 %]; *p* = 1.000).

**Conclusion:**

Afatinib was not associated with cardiac failure or LVEF reductions in the afatinib clinical trial program.

## Background

Afatinib is an oral, irreversible ErbB family blocker that targets the epidermal growth factor receptor (EGFR/ErbB1), human epidermal growth factor receptor 2 (HER2/ErbB2), and HER4 (ErbB4) [1, 2], which results in the inhibition of ErbB3/HER3 phosphorylation [3]. This agent was approved by the US Food and Drug Administration (FDA) in July 2013 for the first-line treatment of metastatic non–small cell lung cancer (NSCLC) in patients whose disease harbors common *EGFR* mutations, namely exon 19 deletions (Del19) or exon 21 (L858R) substitutions, as identified by an FDA-approved test [4]. The first-line, phase III LUX-Lung 3 (LL3) trial in metastatic *EGFR* mutation–positive NSCLC had documented a significant improvement in the primary endpoint of progression-free survival (PFS) with afatinib 40 mg/day versus pemetrexed/cisplatin (11.1 vs 6.9 months; hazard ratio [HR], 0.58; *p* = 0.001) for all patients with *EGFR* mutations [5]. A larger median PFS difference was noted for patients with common mutations (13.6 months for afatinib vs 6.9 months for chemotherapy; HR, 0.47; *p* <0.001) [5, 6]. The LUX-Lung 1 (LL1) trial was a phase IIb/III study in patients with chemotherapy- and EGFR tyrosine kinase inhibitor (TKI)–progressive NSCLC, in which afatinib 50 mg/day prolonged PFS versus placebo (3.3 vs 1.1 months; HR, 0.38; *p* <0.0001), but there was no corresponding improvement in the primary endpoint of overall survival (OS; 10.8 vs 12.0 months; HR, 1.08; *p* = 0.74) [7]. First-line afatinib significantly prolonged OS for patients with Del19 mutations compared with chemotherapy in LL3 and LUX-Lung 6 (HR, 0.54; *p* = 0.0015 and HR, 0.64; *p* = 0.023, respectively) [8]. The approved administration of afatinib is at the 40-mg dose level, to be given orally once daily [4].

The HER2 pathway is known to play a role in normal cardiac function [9]. HER2 is expressed in the heart, and preclinical studies suggest that HER2 downstream pathways are important for cardiomyocyte survival [10]. Therefore, cardiotoxicity is an established safety concern with the use of anticancer agents designed to target this particular pathway, including the anti-HER2 monoclonal antibody trastuzumab [11, 12] and the anti-HER2/EGFR TKI lapatinib [13]. The cardiac adverse event (AE) profile of molecularly targeted agents, including the aforementioned anti-HER2 therapies and multitargeted small-molecule TKIs, such as sunitinib, primarily consists of symptomatic or asymptomatic declines in left ventricular ejection fraction (LVEF) or alterations in blood pressure, with prolongation of the corrected QT interval and cardiac dysrhythmias as additional risks [14–17]. Cardiac AEs reported with these newer agents are typically low-grade and reversible, including LVEF reductions [18, 19] and blood pressure elevations with or without secondary or end-organ involvement [17, 20]. Although trastuzumab- and TKI-attributable high-grade cardiac AEs and cardiovascular mortality are rare, concerns regarding the inherent propensity for cardiovascular AEs have resulted in widespread monitoring and assessment of multiple cardiac parameters in patients receiving these agents. At the same time, however, assessing their true clinical impact has been problematic. Unlike the anthracyclines, for which cardiotoxicity is cumulative and dose related [21], cardiac AEs associated with targeted therapies have been far less predictable, have generally been reversible, and have not precluded reintroduction of these agents once the initial cardiac event has been controlled [18–20]. Given the potential for cardiotoxicity with HER2 inhibitors and the known activity of afatinib against several ErbB family members, including HER2, it is important to establish the cardiovascular AE profile for afatinib as part of the clinical development process.

A phase II trial of afatinib utilized electrocardiograms (ECGs) to assess possible QTcF (QT interval corrected by the Fridericia formula) effects and found no afatinib effect on cardiac repolarization [22]. The analyses reported herein were conducted to further characterize the cardiac safety of afatinib, based on cardiac failure AEs (CF-AEs), including symptomatic cardiac failure and LVEF reductions, reported from LL1 and LL3, as well as patient information available from a large number of additional trials that was derived from the broader clinical databases.

### Patients and methods

#### Study design, patients, and treatment

The study designs of LL1 (phase IIb/III) and LL3 (phase III), both conducted in patients with stage IIIB/IV adenocarcinoma of the lung, are described in detail in their primary publications [5, 7]. In brief, in LL3, EGFR TKI–naive patients with NSCLC and a documented *EGFR* mutation were randomly assigned in a 2:1 ratio to receive either afatinib 40 mg (229 patients) or chemotherapy with pemetrexed/cisplatin (111 patients) [5]. LL1 was conducted in EGFR TKI– and chemotherapy-pretreated (up to 2 lines of chemotherapy) patients, who were randomly assigned 2:1 to receive afatinib 50 mg/day (390 patients) or placebo (195 patients) [7]. In addition to the afatinib-treated patients from LL3 and LL1 (*n* = 619), 3246 additional patients from all 47 completed, Boehringer Ingelheim–sponsored, phase I-III clinical trials of afatinib, as well as from reported clinical experience, were included in the pooled analysis. No trials were excluded except for 1 blinded clinical trial of afatinib in head and neck cancer that included only 3 patients at the time of the data cut. Overall, these trials provided a combined cohort of 3865 patients treated with afatinib, as monotherapy or as a component of a combination regimen. This pooled data analysis set is a heterogeneous population in terms of tumor type, prior therapy, and afatinib dose and treatment.

When feasible, LVEF was assessed at baseline, every 12 weeks, and at the end of treatment. In all trials, AEs were categorized and graded using National Cancer Institute Common Terminology Criteria for Adverse Events (NCI-CTCAE) version 3.0 [5, 7].

All studies were conducted in accordance with the Declaration of Helsinki and guidelines on Good Clinical Practice, and the protocols were approved by local ethics committees at each participating center.

#### Data analysis

The endpoints of interest for this secondary analysis were cardiac failure–related AEs and clinically significant LVEF reductions, per multigated acquisition (MUGA) scan or echocardiogram (ECHO). The Standardized MedDRA Query (SMQ) of “Cardiac Failure” was utilized to identify CF-AEs (eg, acute left ventricular failure, high-output cardiac failure, left ventricular failure) that were reported during the routine AE surveillance of the trials. The SMQ was modified by removing preferred terms considered non-specific (eg, edema) that are common in the target population. Time at risk (TAR)–adjusted CF-AE rates were used to compare treatment arms in LL1 and LL3 due to large differences in treatment exposures. TAR-adjusted CF-AE rates were calculated as events per 100 patient-years, with the treatment groups being compared using proportional hazards regression. Clinically significant LVEF reductions were examined using the following criteria: LVEF <50 and ≥10 % decrease from baseline or LVEF ≥50 and ≥15 % decrease from baseline, with partial recovery defined as an increase in ejection fraction of ≥10 percentage points from the nadir to a level ≤5 percentage points below baseline, and full recovery defined as recovery to within 5 percentage points of baseline. The frequency of clinically significant LVEF reductions in the 2 treatment arms of LL3 and LL1 were compared using 2 × 2 contingency tables and Fisher exact test to determine 2-tailed *p*-values.

## Results

### LL3

The 229 patients who received afatinib 40 mg/day had a mean treatment duration of 11.5 months, during which 5 patients had CF-AEs (*n* = 4; 1.7 %) or an LVEF reduction (*n* = 1; 0.4 %). The comparator group of 111 patients treated with chemotherapy had a substantially shorter mean treatment duration of 3.7 months, during which 1 CF-AE (0.9 %) and no LVEF declines were reported. The TAR-adjusted CF-AE rates (events per 100 patient-years) were similar for afatinib versus chemotherapy (2.28 vs 2.92; Table 1). There was 1 grade ≥3 CF-AE, which was in the chemotherapy group; all other CF-AEs in both arms were of grade 1 or 2.

**Table 1:** TAR–adjusted CF-AE rates in LL3, LL1, and a pooled analysis of 3865 patients

LL3	Afatinib (40 mg/day) *n* = 229	Chemotherapy *n* = 111
TAR (mo)	11.5	3.7
TAR-adjusted CF-AE rate (events/100 patient-years)	2.28	2.92
HR (95 % CI)	1.18 (0.12, 11.39; *p* = 0.8870)	
LL1	Afatinib (50 mg/day) *n* = 390	Placebo *n* = 195
TAR (mo)	5.1	2.8
TAR-adjusted CF-AE rate (events/100 patient-years)	2.40	2.23
HR (95 % CI)	1.32 (0.14, 12.50; *p* = 0.8086)	
Pooled	Afatinib *n* = 3865	
TAR (mo)	5.7	
TAR-adjusted CF-AE rate (events/100 patient-years)	2.88	

*TAR* time at risk, *CF-AE* cardiac failure adverse event, *LL3* LUX-Lung 3, *LL1* LUX-Lung 1, *HR* hazard ratio, *CI* confidence interval

Six CF-AEs were noted in 5 afatinib-treated patients, all of which were associated with a transient decrease in LVEF, and the LVEF recovered toward baseline in 4 patients despite continuation of afatinib therapy; all events were non-serious and low grade (grade ≤2), and 3 events were considered related to study drug (2 cases of diastolic dysfunction and 1 case of left ventricular dysfunction). One patient had grade 2 left ventricular dysfunction 567 days after starting afatinib treatment, with a MUGA scan demonstrating LVEF reduction from 65 % at baseline to 47 % at this time point. Afatinib treatment was discontinued 1 month later, and a follow-up MUGA scan after 1 month showed an LVEF increase to 56 %. Of note, 4 of the 5 patients had multiple cardiac baseline conditions, which included coronary artery disease, hypertension, hypercholesterolemia, tricuspid regurgitation, mitral regurgitation, pericardial effusion, aortic valve sclerosis, and diastolic dysfunction.

**Fig. 1:**
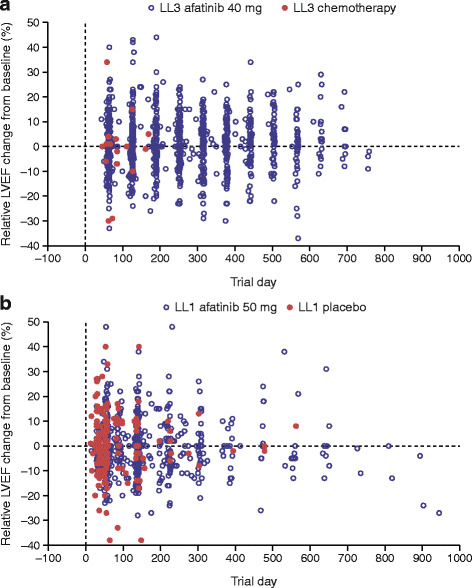
**a** Relative LVEF change from baseline in LL3. Although there was no global tendency to decline, there were 3 LVEF measurements in LL3 that were reduced from baseline, and there were no increases beyond the 700-day mark; these represent 3 distinct patients whose LVEF reductions were not clinically significant (<10 % change from baseline) and were >50 % (the lowest LVEF was 61 %). **b** Relative LVEF change from baseline in LL1. Although there was no global tendency to decline beyond the 700-day mark, 7 LVEF measurements were reduced or at neutral; these 7 LVEF measurements were from 2 patients, and 1 patient had 3 measurements beyond day 700 (76.1 % on day 726, 76.9 % on day 810, and 74.4 % on day 894) that were close to his baseline LVEF of 76.80 %. Another patient had 4 LVEF measurements beyond day 700 (76.3 % on day 735, 65.3 % on day 819, 56.5 % on day 903, and 55.0 % on day 945); disease progression was diagnosed around the time of the final LVEF measurement. Abbreviations: LVEF, left ventricular ejection fraction; LL3, LUX-Lung 3; LL1, LUX-Lung 1

**Fig. 2:**
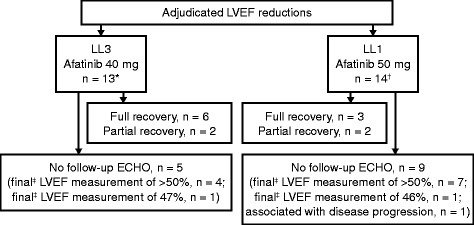
Adjudicated LVEF reductions in the afatinib treatment arms of LL3 and LL1. Six of the 13 patients in the afatinib group for LL3 achieved a full recovery, and 2 patients had a partial recovery. Follow-up ECHOs were not available for 5 patients, 4 of whom had a final LVEF measurement of >50 %. The remaining patient had a final LVEF measurement of 47 %. Three of the 14 patients in the afatinib group for LL1 achieved a full recovery, and 2 patients had a partial recovery. Follow-up ECHOs were not available for 9 patients, 7 of whom had a final LVEF measurement of >50 %. One patient had disease progression and another patient experienced a final LVEF measurement of 46 %. Clinically significant LVEF reductions were examined using established criteria: LVEF <50 and ≥10 % decrease from baseline or LVEF ≥50 and ≥15 % decrease from baseline. Abbreviations: LVEF, left ventricular ejection fraction; LL3, LUX-Lung 3; LL1, LUX-Lung 1; ECHO, echocardiogram; SD, standard deviation. ^*^Mean (SD) percent change from baseline to minimum LVEF during treatment: −24.90 (4.86) for afatinib and −29.30 (0.99) for chemotherapy; mean (SD) percent change from baseline to last LVEF during treatment: −13.95 (10.83) for afatinib and −29.30 (0.99) for chemotherapy. ^†^Mean (SD) percent change from baseline to minimum LVEF during treatment: −22.71 (3.33) for afatinib and −31.24 (7.94) for placebo; mean (SD) percent change from baseline to last LVEF during treatment: −16.88 (8.45) for afatinib and −29.38 (11.52) for placebo. ^‡^Last reported LVEF measurement at database lock

The mean percent change from baseline to last LVEF measurement was similar for the 2 treatment groups: −1.06 % for chemotherapy and +2.17 % for afatinib (Table 2 and Fig. 1a). Of note, only 15 patients in the chemotherapy group had a follow-up LVEF measurement, compared with 208 patients in the afatinib group. Based on the criteria for defining clinically relevant LVEF reductions, clinically significant LVEF reduction occurred in 13 of 208 (6.3 %) patients in the afatinib 40-mg group and 2/15 (13.3 %) patients in the chemotherapy group, but the rates were not statistically significant between the 2 groups (*p* = 0.267). LVEF data are summarized in Table 2 and further summarized for the 13 afatinib-treated patients with clinically significant LVEF reductions in Fig. 2.

**Table 2:** Descriptive statistics for LVEF by treatment^a^

LL3	Afatinib (40 mg/day) *n* = 229	Chemotherapy *n* = 111
Baseline LVEF, mean (SD)	66.0 (6.90)	66.60 (8.0)
Minimum LVEF during treatment, mean (SD)	62.63 (6.33)	68.87 (10.59)
% change from baseline to minimum, mean (SD)	−4.90 (9.95)	−1.06 (15.30)
Last LVEF during treatment, mean (SD)	67.30 (7.10)	68.90 (10.60)
% change from baseline to last, mean (SD)	2.17 (11.41)	−1.06 (15.30)
LL1	Afatinib (50 mg/day) *n* = 390	Placebo *n* = 195
Baseline LVEF, mean (SD)	64.90 (6.60)	63.90 (7.70)
Minimum LVEF during treatment, mean (SD)	62.35 (6.57)	63.73 (7.87)
% change from baseline to minimum, mean (SD)	−3.41 (9.14)	−0.88 (12.13)
Last LVEF during treatment, mean (SD)	64.20 (7.0)	64.40 (7.70)
% change from baseline to last, mean (SD)	−0.39 (10.40)	0.27 (12.62)

*LVEF* left ventricular ejection fraction, *LL3* LUX-Lung 3, *SD* standard deviation, *LL1* LUX-Lung 1, *ECHO* echocardiogram

^a^In LL3, 208 patients in the afatinib arm and 15 patients in the chemotherapy arm had a follow-up ECHO; in LL1, 304 patients in the afatinib arm and 122 patients in the placebo arm had a follow-up ECHO

### LL1

For afatinib 50 mg/day, during a mean treatment duration of 5.1 months, 4 of 390 patients (1.0 %) had a CF-AE. Three of 4 events were assessed by the investigator as unrelated to afatinib, with alternative etiologies specified as follows: grade 3 acute left ventricular failure (secondary to a hypertensive crisis), grade 4 cardiac failure (concurrent pneumonia), and grade 5 cardiac failure attributed to disease progression. The remaining CF-AE in the afatinib group was a fatal acute left ventricular failure, assessed by the investigator as treatment related. This event occurred in a 60-year-old female with a history of chronic obstructive pulmonary disease, emphysema, and chronic renal insufficiency, who was hospitalized for a severe pulmonary infection 4 weeks after starting afatinib. The next day, the patient’s brain natriuretic peptide level was 10,167 pg/mL (normal range, 0–336 pg/mL) and a clinical diagnosis of acute left heart failure was made. Due to the patient’s critical condition, diagnostic imaging, such as ECHO, computed tomography scan, or chest x-ray, was not performed. The patient died 4 days later, but no autopsy was performed. The comparator group of 195 placebo recipients had a shorter mean treatment duration of 2.8 months, during which 1 patient experienced cardiac failure (0.5 %). There were no LVEF reductions reported as CF-AEs in either group. The TAR-adjusted CF-AE rates were similar for afatinib versus placebo (2.40 vs 2.23; Table 1). All the CF-AEs in both groups were of grade ≥3 severity.

The mean percent change from baseline to last LVEF measurement was comparable between groups (+0.27 % placebo, −0.39 % afatinib; Table 2 and Fig. 1b). LVEF data are summarized in Table 2 and further summarized for the 14 afatinib-treated patients with clinically significant LVEF reductions in Fig. 2. The frequency of clinically significant LVEF reductions for afatinib was similar to placebo (14/304 [4.6 %] vs 5/122 [4.1 %]; *p* = 1.000). There was no uniformity in the severity or the time to onset of the LVEF reductions.

### Pooled analysis

The 3865 patients who received afatinib (any dose) in the pooled analysis had a mean duration of treatment of 5.7 months, during which 53 (1.4 %) patients developed CF-AEs, the majority of which were grade ≤2. The TAR-adjusted CF-AE rate for this analysis population was 2.88 (Table 1), which is consistent with that seen with afatinib in LL1 and LL3.

## Discussion

The current analysis, capturing data from 2 controlled phase III trials in NSCLC and a pooled analysis of 49 trials conducted in various settings, supports low rates of protocol-defined cardiac failure and clinically significant LVEF reductions for the ErbB family blocker afatinib that are comparable to those seen with the control treatment arms. These results indicate that afatinib was not associated with cardiac failure or clinically significant LVEF reductions in this clinical trial program.

The established cardiotoxic potential of anti-HER2 therapeutics, such as trastuzumab [11, 12] and lapatinib [13], as well as multitargeted TKIs, such as sunitinib [20], has heightened the importance of evaluating the cardiac safety profile of new targeted agents. In our analysis of afatinib reported here, to ensure that events indicative of cardiac failure were not underestimated, detailed analyses were designed to identify patients experiencing cardiac failure based on a modification of the MedDRA SMQ. In addition to cardiac failures, clinically significant decreased LVEF readings via MUGA scan or ECHO were also analyzed. In the reporting of LVEF reductions, we opted for the application of conservative criteria in defining clinically relevant events. Given the likelihood of low frequencies of CF-AEs in the individual trials in NSCLC, the overall cumulative pool of patients (*n* = 3865) receiving any afatinib dose (as monotherapy or in combination) was also analyzed to provide a larger data set. Results for CF-AEs in this pooled analysis confirmed the results observed for afatinib 40 mg in LL3 and afatinib 50 mg in LL1. The frequency of CF-AEs in the pooled analysis (1.4 %) was consistent with those for the afatinib groups of the randomized controlled trials (2.2 and 1.0 % in LL3 and LL1, respectively). With exposure correction, the TAR-adjusted CF-AE rates were likewise similar across the 3 groups of afatinib recipients: 2.88 in the pooled analysis versus 2.28 in LL3 and 2.40 in LL1 (Table 1). The incidence of CF-AEs with maximum CTCAE grade ≥3 in the pooled analysis (0.5 %) was also within the range of that for the individual studies (0.0 and 1.0 % in LL3 and LL1, respectively).

Cardiovascular AEs related to anticancer treatment fall into 2 distinct groups, generally referred to as Type I (resulting in dose-related toxicity with destruction of myocytes) and Type II (for which myocyte dysfunction without characteristic biopsy changes of myocyte destruction is a cardinal characteristic) [23]. Whereas anthracyclines are known to produce Type I cardiotoxicity, trastuzumab, sunitinib, and other targeted agents have been associated with Type II characteristics [24]. This is a clinically important distinction, as reintroduction and prolonged treatment with a Type II cardiotoxic agent may be feasible in many cases [19]. Based on the current analyses, afatinib does not appear to be associated with significant toxicity of either the Type I or Type II form. Furthermore, preclinical and clinical data indicate that afatinib does not have an effect on the QTc interval [22]. The TAR-adjusted CF-AE rates for afatinib did not vary significantly from those of the comparator or control groups. However, it is well appreciated that cardiotoxicity may be subclinical, with a large number of patients required to detect small changes in cardiac parameters. No global subclinical cardiac changes were observed in the current analysis, and there was a lack of the characteristic clustering of cardiac events that has been observed with known cardiotoxic agents [25]. Overall, in our evaluation of clinical trial data for afatinib, we identified no consistency in the nature of the rare cardiovascular events and no evidence of direct or primary cardiotoxicity. As with other agents, it is possible that afatinib may increase the likelihood of cardiovascular AEs in patients with limited reserves.

To date, no observational studies of baseline and on-treatment rates of cardiac failure, specifically in patients with *EGFR* mutation–positive NSCLC, have been published. Prior studies in patients with NSCLC, without regard to *EGFR* mutational status, had illustrated a high rate of cardiovascular comorbidity in this population (approximately 20 to 36 %, depending on the definitions used) [26–29], as well as increased cardiac failure rates in chemotherapy-treated versus -untreated patients [30, 31]. In an analysis of the US SEER–Medicare database that included 34,209 patients aged ≥65 years with NSCLC (with follow-up through 2005), the risks of cardiac failure and dysfunction were increased by 29 and 58 %, respectively, among patients receiving chemotherapy (without concurrent radiation) relative to those receiving no chemotherapy or radiation [31]. Moving forward, additional analyses will be important for defining the cardiac risk specific to TKI therapy for *EGFR* mutation–positive NSCLC.

We acknowledge that there are limitations inherent to our analysis, which was of a post hoc nature and based on data from clinical trials for which characterizing the cardiac AE profile of afatinib was not the primary objective. The treatment durations for afatinib were relatively short, measured in months rather than years, with long-term safety assessments not currently possible for malignancies such as advanced NSCLC (for which survival durations are still limited). Follow-up ECHOs were performed in the majority of patients, but in some clinical situations, it was determined by the investigator to be infeasible. In addition, while the results of the pooled analysis were consistent with those from the individual randomized trials and provide some reassurance of a low cardiotoxic potential for afatinib, the pooled data need to be interpreted with a particularly high level of caution given that LVEF monitoring was not consistent in many of these trials and there was variability with respect to other aspects of the study designs (including the use of afatinib as monotherapy or as a component of combination regimens). Furthermore, as patients with baseline abnormal cardiac function were excluded from these trials, the effect of afatinib on this population remains unknown. Clearly, additional studies are needed to further characterize the risk of TKI-associated cardiotoxicity in patients with advanced NSCLC and any potential cardiac AE profile nuances for approved and investigational TKIs.

## Conclusions

In conclusion, afatinib shows no evidence of direct or primary cardiac or cardiovascular toxicity. However, rare events in vulnerable patients who have limited cardiac reserves could not be ruled out. Experience with agents that manifest cardiovascular toxicity suggests that minimizing cardiac stress prior to treatment may mitigate the expression of such events, and monitoring of patients deemed to be at high risk of cardiovascular events is prudent.

## Abbreviations

AE: adverse event
CF: cardiac failure
CI: confidence interval
ECG: electrocardiogram
ECHO: echocardiogram
EGFR: epidermal growth factor receptor
HER2: human epidermal growth factor receptor 2
HR: hazard ratio
LL1: LUX-Lung 1
LL3: LUX-Lung 3
LVEF: left ventricular ejection fraction
MUGA: multigated acquisition
NCI-CTCAE: National Cancer Institute Common Terminology Criteria for Adverse Events
NSCLC: non–small cell lung cancer
PFS: progression-free survival
SD: standard deviation
SMQ: Standardized MedDRA Query
TAR: time at risk
TKI: tyrosine kinase inhibitor
